# Diffusion of Cardiopulmonary Resuscitation Training to Chinese Immigrants with Limited English Proficiency

**DOI:** 10.1155/2011/685249

**Published:** 2011-02-21

**Authors:** Mei Po Yip, Brandon Ong, Shin Ping Tu, Devora Chavez, Brooke Ike, Ian Painter, Ida Lam, Steven M. Bradley, Gloria D. Coronado, Hendrika W. Meischke

**Affiliations:** ^1^Division of General Internal Medicine, School of Medicine, University of Washington, 325 9th Avenue, Seattle, WA 98104, USA; ^2^Department of Health Services, Northwest Center for Public Health Practice, 1107 NE 45th Street, Seattle, WA 98105, USA; ^3^Family and Youth Services, Chinese Information Service Center, 611 S Lane Street, Seattle, WA 98104, USA; ^4^Public Health Sciences, Fred Hutchinson Cancer Research Center, 1100 Fairview Avenue N, Seattle, WA 98109, USA

## Abstract

Cardiopulmonary resuscitation (CPR) is an effective intervention for prehospital cardiac arrest. Despite all available training opportunities for CPR, disparities exist in participation in CPR training, CPR knowledge, and receipt of bystander CPR for certain ethnic groups. We conducted five focus groups with Chinese immigrants who self-reported limited English proficiency (LEP). A bilingual facilitator conducted all the sessions. All discussions were taped, recorded, translated, and transcribed. Transcripts were analyzed by content analysis guided by the theory of diffusion. The majority of participants did not know of CPR and did not know where to get trained. Complexity of CPR procedure, advantages of calling 9-1-1, lack of confidence, and possible liability discourage LEP individuals to learn CPR. LEP individuals welcome simplified Hands-Only CPR and are willing to perform CPR with instruction from 9-1-1 operators. Expanding the current training to include Hands-Only CPR and dispatcher-assisted CPR may motivate Chinese LEP individuals to get trained for CPR.

## 1. Introduction

In the United States, out-of-hospital cardiac arrest continues to be an important public health problem. Cardiopulmonary resuscitation (CPR), in use for fifty years, is the most effective intervention for pre-hospital cardiac arrest [1–4]. Being an integral part of the “chain of survival,” high-quality CPR can improve out-of-hospital survival rate [5]. CPR can be effectively taught to lay persons as an intervention for out-of-hospital cardiac arrest to initiate resuscitation and “buy time” in the early minutes after cardiac arrest before the arrival of emergency medical services. It is estimated that one life is saved for every 24–36 persons who receive bystander CPR [2, 6].

Public CPR training and instruction has been offered to the general public in different ways. Most notably are formal classes conducted by the American Heart Association and the Red Cross, fire departments, workplaces, schools, and dispatcher-assisted CPR delivered by 9-1-1 operators at the time of the cardiac arrest. Despite all of these available opportunities, the proportion of citizens trained to perform CPR is small and many are unfamiliar with bystander CPR [7–9]. Studies have shown that CPR training does not reach desirable target populations in large numbers [10–12]. The recent new guidelines provided by the American Heart Association to include Hands-Only CPR expands current strategies to disseminate CPR training [13]. Modification of CPR by eliminating mouth-to-mouth ventilation, which is often perceived to be difficult to perform and, for some, possibly embarrassing, may make CPR more accessible to the public and encourage more people to get trained. 

One of the goals of CPR training is to increase the number of citizens capable of performing CPR, and thus increase the rate of bystander CPR. However, surveillance data have also identified certain communities that have persistently low rates of bystander CPR [14]. Studies have demonstrated a racial difference in those receiving bystander CPR. In a study investigating racial differences in out-of-hospital cardiac arrest, less bystander-initiated CPR may contribute to the difference in survival between black and white victims [15, 16]. Hispanics are less likely to receive bystander-initiated CPR than non-Hispanics [17]. Latinos, when compared to African Americans and Caucasians, have the lowest rate of receiving bystander CPR [18]. The bystander CPR rate in Asians has not been reported. However, in a study to understand socioeconomic factors influencing bystander CPR, neighborhoods with increased percentages of Asian residents and linguistically isolated households were associated with lower rates of bystander CPR [19]. Most people trained in CPR are more likely to be from younger age groups, higher socioeconomic status, higher education levels, and speak English as a first language [10, 20–22]. Weiner et al. reported disparities in the knowledge of basic life support between whites and Hispanics [22]. A recent study indicated that Asian Americans have lower rates of CPR training than whites [23].

Nationally, among Asian-American subgroups, Chinese Americans have the highest proportion of individuals reporting speaking English less than “very well” [24]. Forty-five percent of the Chinese population living in Washington State reported they “spoke English less than very well” [25]. Access to CPR training, which is usually conducted in English, is likely to be less available to limited English proficiency (LEP) communities. People with limited skills in English have difficulty understanding written and verbal information and, therefore, are not often reached by English educational materials or media-based campaigns using video, Internet, or television [26]. We have not found any published studies investigating the diffusion and dissemination of CPR training in LEP communities.

**Theoretical Framework**

**Figure 1:**
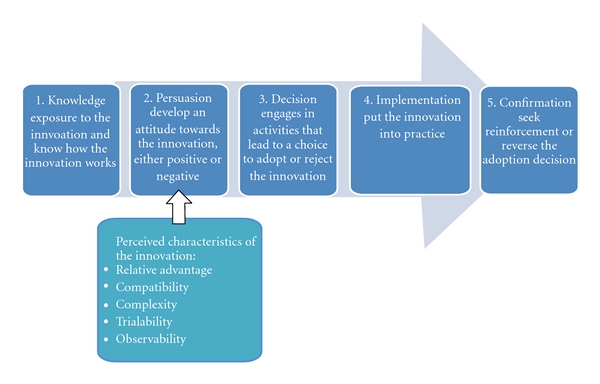
Five stages in the innovation-decision process.

This study is guided by Roger's theory of diffusion. Diffusion is the process in which an innovation is communicated through certain channels over time among the members of a social system [27]. According to Rogers, an innovation is an idea, practice, or object that is perceived as new by an individual. It does not matter whether or not that an idea is “objectively” new as measured by the lapse of time since its first discovery [27]. The innovation-decision process is an information-seeking and information-processing activity in which an individual learns about the innovation and makes a decision to adopt or reject it. As described by Rogers, it is sequential and occurs over time, consisting of a series of different actions (Figure 1). Information exchange occurs in the five stages among members of the social system through different mass media, interpersonal communication networks, and interactive communication. An individual evaluates the innovation based on the new information, shared attitudinal influences, and need for behavioral change.

Knowing the existence of innovation is the first stage of the innovation-decision process. With respect to CPR, an individual in this stage learns about “innovation” (i.e., CPR) either because of a need (e.g., job requirement) or passively (e.g., passing by a billboard which promotes CPR). “Persuasion” is the second stage of the process in which an individual develops a favorable or unfavorable attitude toward the innovation. Unlike the first stage which focuses on “knowing” the innovation, the persuasion stage focuses on how an individual “feels” about the innovation. At this stage, perceived characteristics of the innovation determine the rate of adoption. These characteristics include relative advantage, compatibility, complexity, trialability, and observability [27].

In this study, we examine attributes of the innovation-decision process that influence the adoption or rejection of CPR. For example, we would like to understand how the concept of CPR is introduced to LEP individuals. How do Chinese LEP perceive CPR? What are the factors influencing decisions to get trained? Where and how do Chinese LEP obtain information about CPR training? Are Chinese LEP receptive to performing CPR with instruction? Have Chinese LEP heard of Hands-Only CPR?

## 2. Materials and Methods

### 2.1. Recruitment

We conducted five gender-matched focus groups in Mandarin and Cantonese between December 2009 and April 2010. Adult Chinese LEP men and women living in Seattle, Washington were recruited by convenience sampling. The use of focus groups is an effective qualitative method to seek opinions, values, and beliefs in a collective context that provides insights into participants' opinions and experiences through group interaction [28, 29]. They have also been used with culturally and linguistically diverse populations [30–32]. Participants were recruited from the Chinese Information and Service Center (CISC), a community-based organization that provides cultural orientation and social services to immigrants. Chinese adults aged 18 or above who self-reported speaking English “not well” or “not at all” were invited to participate in the study. We welcomed individuals who had or had not received CPR training to participate in the discussion. The goal of recruitment is to achieve homogeneity in background (i.e., Chinese LEP) rather than attitude (i.e., toward CPR training). Participants signed informed consent forms and provided basic demographic data prior to the focus group discussions. Participants were offered an honorarium as a token of appreciation for their time and were served light refreshments. All focus groups were conducted in the participants' native dialect. Each focus group consisted of 8–10 participants and the discussion lasted for about 1.5 hours.  Table 1 shows an example of questions based on the innovation-decision process.

The first author, who is bilingual in English and Cantonese, conducted all focus groups with the assistance of a trilingual interpreter (English, Mandarin, and Cantonese). The discussion started off with a question “Have you heard of CPR?” If all participants were not aware of CPR, the facilitator explained by saying “Cardiopulmonary resuscitation (CPR) is an emergency life-saving method to restart a person's heart. It consists of manually pushing up and down on a person's chest to pump their heart until more advanced medical care is available.” The facilitator invited those who had been CPR trained to share the meaning of CPR to the group. We also showed a short video clip of Hands-Only CPR to help participants visualize the CPR procedures. All focus group discussions were tape recorded, translated, and transcribed verbatim. An independent trilingual translator (English, Mandarin, and Cantonese) first listened to the Chinese audio recording multiple times and then performed the translation and transcription. Transcripts were checked against the tapes for accuracy.

Transcripts were analyzed by content analysis guided by the innovation-decision process. The development of the initial code book was based on these five stages and the perceived characteristics of innovation. Two members of the research team independently reviewed the transcript and applied the codes to phrases written in the transcript. The reviewers then met to discuss the content of each transcript, codes applied, and any new information. Differences of opinion were resolved through discussion and consensus. The codes were then compared and similar codes were clustered to form categories. Coding categories were refined until all data were coded into exhaustive and exclusive categories [33].

## 3. Results and Discussion

### 3.1. Study Group Characteristics

Forty-six men and women participated in our study. All were born outside the US first-generation immigrants with nearly two-thirds having lived in the US less than 10 years. More than 60% of the study participants had attained more than 10 years of education but only one-third is currently employed. Most participants were married and lived with family.  Table 2 shows the demographics of the study participants. 

### 3.2. CPR Knowledge

To our surprise, the majority of participants were not familiar with CPR. When introduced, participants demonstrated some understanding of the basic CPR concept. For example, the majority identify when CPR is needed and that CPR can save lives. However, no one was able to describe how CPR works. Of all the participants, only six (13%) were CPR trained. Half of them received training because of job requirements whereas the others received training at a church or a community-based organization for various reasons. CPR was offered and made available to participants rather than the individual actively pursuing opportunities to get trained. Except for those who got trained, none could name organizations commonly known to offer CPR classes, like the Red Cross. The unpopularity of CPR among the LEP Chinese community was described by a participant:

“I think many Chinese here do not know how to call 9-1-1 and they don't know what to expect from 9-1-1 [dispatcher]. Also, most of us don't know what CPR is. High school students, Chinese immigrants for those who are in their thirties to fifties don't know. They have never learned that [CPR]. I think over 80–90% of us don't know what is CPR. If we can make it more popular, it would be good to every one of us.”

### 3.3. Relative Advantage of Performing CPR

Even though, to most participants, being a good samaritan is consistent with their values and beliefs, many would hesitate to actually perform CPR in an emergency. Some participants preferred calling 9-1-1 to starting CPR. Participants perceived greater benefits of calling 9-1-1 than performing bystander CPR which would result in a better outcome. Participants are not confident in performing CPR. Worries about doing CPR improperly, which could cause further harm, and the lack of understanding of the role of CPR in the chain of survival impedes LEP individuals from initiating CPR. Focus group participants have different views on what should be done:

Participant A asked:
“Is dialing 9-1-1 [versus performing CPR] the better way to do?”
Participant B replied:
“Sometimes it may be too late [delay performing CPR].”
“It is true.” agreed participant C.
Participant D objected, “I think we should call 9-1-1 first. Ya … the best way to do is to call 9-1-1. If that person has no response and you perform CPR on him, he may accuse you of wrong doing after he regains his conscious. I just afraid of situation like this. That's my worry. I think calling 9-1-1 is the best way.”
“Yes. Call 9-1-1 first since we are not professional. At least we are covered. Then we try our best to help. I think this is the way to go,” said participant D.
Participant E responds, “Yes. I never learn and don't know what to do. I have no way to offer help. I will see if there is anyone stop by and ask for his assistance. If there is no one around, I just wait until 9-1-1 people come.”

### 3.4. Persuasion: Favorable Attitude toward CPR Certification

With only a handful of participants trained, many of the discussions were carried on by those who were not familiar with CPR training. When told, many participants favored the idea of certification. They felt *being certified* would protect them against possible legal liability in the future and that certification *permits* the bystander to perform CPR and that they are *qualified* to do so. Participants felt they were more likely to perform CPR if they are certified. The issue of certification is revealed through the discussion:

Participant A:
“At least we showed some knowledge [trained for CPR]. Besides [learning the skill], getting a card or piece of paper, writing down that you had completed the course. If worst comes to worst, you have the card to prove you have been trained.”
The other participant responds, “Yeah, I feel more confident. It makes a difference. Here in US we talked about the certificate all the time. Even an electrician has a certificate [license]. It would make me feel more comfortable and confidence doing CPR.”
Puzzled, one participant asked “I want to ask if one does not have the card-can we still perform CPR?”

### 3.5. Complexity of CPR Procedure

The two biggest challenges confronting LEP participants who have not been trained are the procedure itself and the possibility of causing further harm. Some participants felt rescue breaths are more difficult to perform than chest compression. Others were not comfortable performing rescue breathing, particularly to a stranger. Several female participants felt embarrassed performing the mouth-to-mouth breathing on a male victim. Others worried about hygiene and the possibility of contracting infectious diseases. As for chest compressions, there were concerns about breaking the victim's ribs when pushing too hard, especially with children or the elderly:

one participant said,
“I dare not to offer help [doing CPR] as I might break his rib. You may make things worse. This is not good.”
Another participant elaborates further,
“Maybe he will recover [without intervention]. But because you do the compression incorrectly, you may actually cause further harm. You may even cost his life! It makes me feel bad if that happens.”

Many participants felt it is important to master the CPR skill. Competency is critical for a participant to apply CPR confidently in the future. Participants are eager to learn correct hand position, how to locate the heart, depth of chest compression, as well as of compression rate. To be competent, practice is the key:

Participant A:
“We must practice. We can't just learn by watching.”

Perhaps the rationale to stress practice is because CPR is a life saving measure; therefore, one should take it seriously and diligently. As one participant said:

“We have to master the key points. Learning without practice means nothing. You have to master the skill well since it is a life-and-death issue. Otherwise, how can you do that right [to the victim]? You don't have the confidence. One have to [make sure] master the skill first before rescue other people.”

### 3.6. Process of Implementation: Learning CPR through Dispatcher Instruction and Hands-Only CPR

Other ways to learn CPR is through dispatcher instruction when calling 9-1-1 or through the recently introduced Hands-Only CPR, a simple way to perform this life saving skill. In general, participants welcomed both alternative methods. One participant said,

“I think it does not matter if the method you use [standard versus Hands-Only CPR]. We just use whatever method that is more effective to save people's life.”

There are advantages to each method. Hands-Only CPR was accepted by the majority of participants because of it does not involve rescue breathing. One participant said,

“It [Hands-Only CPR] reduces the embarrassment of mouth-to-mouth ventilation. We don't have to be afraid of [mouth-to-mouth ventilation]. It is more convenient.”
Agreed by another participant,
“It is more hygienic. You don't need to blow with your mouth.”

Dispatcher-assisted CPR provides another opportunity for individuals who had never received training to perform CPR. With guidance, many participants said they are willing to do CPR. However, participants feel more confident in performing CPR if they have prior knowledge. A participant shared,

“I am sure I will be very nervous. I will try to keep calm. I will press the speaker phone so I do not need to hold the phone, it will waste my time. I will listen and follow his instruction to do CPR. I will try. I wish I could help. It is better than not helping.”

Participant also shared what the dispatcher can do to help perform CPR:

“If you guide me going through the process [CPR] which I have no idea, the first thing to do is that you must speak my language. Then, you have to explain to me what we will be doing, why we are doing so and what we are going to achieve. I will feel confidence to follow if you tell me these. If you just tell me what to do without telling me why, I don't know whether I am doing it right or not. You tell me step by step and I will follow. Just like a student follows the teacher's instruction:simple, step by step.”

### 3.7. Decision to Learn CPR: The Motivation Behind It

Few participants in our focus group had seriously considered getting trained for CPR. The majority of the participants who had been trained received training as a job requirement. Only one participant said she voluntarily went to training because of her aging husband. Many thought there was no strong urge or incentive to learn CPR. Time, cost, and language barriers are other factors affecting LEP individuals' intention to receive training. For those interested, getting prepared is the greatest motivation.

“I think the more you learn, the better you will be. Especially the knowledge [CPR] we talk about. We may come across this anytime, anywhere then we can apply what we had learned [CPR] to help other people. If we don't know about this [CPR], there is no way I can help,” said one participant.
“Though I never use it, but at least I know how to respond in times of need,” another participant replied.
Another participant who had received training said, “Well, when it comes to reality [situation that require him to perform CPR]. I am not sure whether I will help. At that time [enroll in CPR training], I thought this will benefit my family in emergency. I don't know what is CPR but thought it might be good to learn.”

## 4. Conclusions

This is the first qualitative study to explore issues related to diffusion of CPR training in Chinese LEP communities. Although that the majority of participants are willing to offer help in an emergency, many simply have not heard of this life-saving measure. Our study findings contribute to the opportunity to further disseminate CPR to the LEP Chinese community. In order to promote diffusion of CPR training, more work is needed to communicate with LEP (1) to increase CPR knowledge, (2) to promote Hands-Only CPR, (3) and to provide appropriate resources and channels. 

Several attributes of the innovation-decision process related to the adoption of CPR deserve consideration. First, more education is needed to improve LEP person's understanding of CPR to narrow the existing knowledge gap. Basic concepts like definition, functions, and principles of CPR need to be introduced. In particular, the impact of early CPR on survival needs to be emphasized. Performing CPR together with calling 9-1-1 can be framed as an effective strategy to save a life of a family member, a situation that often happens at home. This may help in creating a need to motivate individuals to learn CPR as it is relevant and potentially useful in their daily lives. It is also important for LEP individual to realize that for those who suffered from cardiac arrest, their situation would not be made worse by poor CPR. Misconception of the good Samaritan law needs to be corrected to alleviate LEP Individual's concern of legal liability, that it is worth it to get trained. To include good Samarian law as one of the topics in the CPR class may reduce bystanders' hesitation to assist, for fear of being sued or prosecuted for “wrongdoing”. Community-based organizations where LEP Individuals congregate may be an alternative venue to conduct CPR classes. CPR can be taught by trained bilingual staff or with an interpreter.

The complexity of innovation is negatively related to rate of adoption. Similar to previous study findings, our participants also acknowledged ventilation breathing is difficult to perform. To promote adoption, this concern needs to be addressed. With the introduction of simplified CPR, it is possible that individuals may become more interested and seek additional information about the reinvention. Hands-Only CPR is more likely to be accepted by lay persons because of the simple instructions. Studies also reported lay persons are more able to perform chest compressions appropriately than conventional CPR [34, 35]. Connecting individuals with appropriate resources and channels which provide related information is vital. Currently, simplified CPR training on the internet or as cell phone applications are free and readily available, thus providing additional resources to expand diffusion of CPR training and to disseminate knowledge of Hands-Only CPR [36]. 

Mass media channels such as radio, television, and newspapers are particularly useful at the knowledge stage to spread information to a large audience rapidly. Messages sent by mass media stimulate the audience to think about learning CPR. Interpersonal channels work in persuading individual to adopt the idea. With the majority of communication channels in English, the use of media in the LEP individuals' native languages may be helpful to spread CPR knowledge broadly and adequately. Alternative interpersonal strategies to accelerate adoption of CPR include using a trained peer to persuade nontrained LEP individuals through modeling or through system changes. It is encouraging that LEP individuals are receptive to performing CPR through dispatchers who speak their native language. LEP may consider a dispatcher a credible source and that performing CPR in the situation is necessary. With dispatchers instructed in CPR, the process of the innovation-decision process related to CPR is shortened. Adoption of CPR can be greatly increased by dispatcher-assisted telephone CPR, which can lead to a 50% increase in bystander rate [37]. Simulation studies demonstrate that CPR instructions delivered by cell phone improves the quality of bystander CPR, although it took longer to initiate the compression [38, 39]. Comments made by focus group participants regarding providing simple rationales for each step by the dispatcher actually helps LEP to individuals perform the procedure. More research needs to be done to investigate the success of real-time instructions.

## 5. Limitations

Several limitations of this study are worth considering. First, we cannot generalize our finding because of the convenience sampling. Our sample consists of a higher percentage of female participants than the demographic profile of Seattle's Chinese population (63% versus 54%) reported in the 2009 American Community Survey. However, we were able to include both trained and nontrained LEP participants in the focus groups because, we believe, participants are interested in the topic. Second, as most participants had not heard of CPR, their perception of CPR may largely be based on viewing the video or from the sharing of others who had trained. Although we do not think the simple, short CPR video clip will create bias in responding, we have not assessed participants' level of comprehension.

## Figures and Tables

**Table 1:** Examples of focus group questions based on the innovation-decision process.

Constructs	Questions
Knowledge	(1) Do you know what CPR is? (2) Are you trained with CPR? (3) Would you know if a person needed CPR?
Persuasion	(4) Does whether or not you would perform CPR depend on the particular situation?
Perceieved characteristics of innovation	(5) What motivates you to get trained for CPR? (6) What are your feelings towards CPR? (7) Are any of your friends or family members CPR trained?
Decision	(8) Do you know where to go to learn CPR? (9) What way of learning CPR sounds best to you?
Implementation	(10) If you were to call 9-1-1 for help and you said that someone wasn't breathing, the emergency operator would give you directions for doing CPR. What could the 9-1-1 dispatcher say that would motivate you to do CPR? (11) Have you heard of “Hands-Only” CPR?

**Table 2:** Study population characteristics (*n* = 46).

*Sociodemographic variables*		*n *(%)
Gender	Male	17 (37%)
	Female	29 (63%)
Age	≥65	15 (37%)
	<65	31 (67%)
Marital Status	Single	3 (7%)
	Married	38 (83%)
	Widow	2 (4%)
	Divorced	1 (2%)
	Other	2 (4%)
Place of birth	China	44 (96%)
	Taiwan	2 (4%)
Length of time living in US	≥10	11 (24%)
	<10	35 (76%)
Years of education	≥10	28 (61%)
	<10	18 (39%)
Employment	Full-time	9 (20%)
	Part-time	6 (13%)
	Unemployed	30 (65%)
	Retired	1 (2%)
Live with family/friends/relatives	Yes	42 (91%)
	No	4 (9%)
Prior experience calling 9-1-1	Yes	7 (15%)
	No	39 (85%)
CPR trained	Yes	6 (13%)
	No	40 (87%)
